# MMP-independent role of TIMP-1 at the blood brain barrier during viral encephalomyelitis

**DOI:** 10.1042/AN20130033

**Published:** 2013-11-26

**Authors:** Carine Savarin, Cornelia C. Bergmann, David R. Hinton, Stephen A. Stohlman

**Affiliations:** *Department of Neurosciences NC-30, Lerner Research Institute, The Cleveland Clinic Foundation, 9500 Euclid Avenue, Cleveland, OH 44195, U.S.A.; †Department of Pathology, Keck School of Medicine, University of Southern California, Los Angeles, CA 90033, U.S.A.

**Keywords:** CD4 T cells, coronavirus, glia limitans, matrix metalloproteinase, TIMP-1, BBB, blood brain barrier, CNS, central nervous system, EAE, experimental autoimmune encephalitis, IFNγ, interferon γ, mAb, monoclonal antibody, MHV, mouse hepatitis virus, MMP, matrix metalloproteinase, PFA, paraformaldehyde, TIMP, tissue inhibitor of matrix metalloproteinases, WT, wild-type

## Abstract

Infection of the CNS (central nervous system) with a sublethal neurotropic coronavirus (JHMV) induces a vigorous inflammatory response. CD4^+^ and CD8^+^ T cells are essential to control infectious virus but at the cost of tissue damage. An enigma in understanding the contribution of T cell subsets in pathogenesis resides in their distinct migration pattern across the BBB (blood brain barrier). CD4^+^ T cells transiently accumulate within the perivascular space, whereas CD8^+^ T cells migrate directly into the CNS parenchyma. As MMPs (matrix metalloproteinases) facilitate migration across the glia limitans, specific expression of the TIMP (tissue inhibitor of MMPs)-1 by CD4^+^ T cells present in the perivascular cuffs suggested that TIMP-1 is responsible for stalling CD4^+^ T cell migration into the CNS parenchyma. Using TIMP-1 deficient mice, the present data demonstrate an increase rather than a decrease in CD4^+^ T cell accumulation within the perivascular space during JHMV infection. Whereas virus control was not affected by perivascular retention of CD4^+^ T cells, disease severity was decreased and associated with reduced IFNγ (interferon γ) production. Moreover, decreased CD4^+^ T cell recruitment into the CNS parenchyma of TIMP-1 deficient mice was not associated with impaired T cell recruiting chemokines or MMP expression, and no compensation by other TIMP molecules was identified. These data suggest an MMP-independent role of TIMP-1 in regulating CD4^+^ T cell access into the CNS parenchyma during acute JHMV encephalitis.

## INTRODUCTION

Immune responses to infections of the CNS (central nervous system) need to be highly regulated in order to limit tissue damage, which could lead to detrimental and even fatal consequences as observed during many neuroinflammatory disorders including multiple sclerosis and viral encephalitis. To control immune function, the CNS displays specialized features, which include lack of lymphatic drainage, parenchymal dendritic cells and MHC (major histocompatibility complex) expression, as well as the presence of the BBB (blood brain barrier) (Galea et al., 2007; Ransohoff and Engelhardt, 2012).

The BBB is a complex and unique structure of the CNS, which controls leukocyte infiltration into the parenchyma (Bechmann et al., 2007). Leukocyte migration from the blood into the CNS parenchyma at the post-capillary venules is a multistep process (Owens et al., 2008). First, immune cells cross an endothelial cell layer associated with a basement membrane to reach the perivascular space. This initial step involves a succession of interactions between leukocytes and the BBB endothelium regulated by adhesion molecules, chemokines and their receptors (Engelhardt and Ransohoff, 2012). Nevertheless, leukocyte accumulation within the perivascular space is not sufficient to exert effective or detrimental responses during CNS infection or neuroinflammatory disorders (Tran et al., 1998; McCandless et al., 2006; Toft-Hansen et al., 2006). To access the CNS parenchyma, leukocytes must also cross the glia limitans composed of another basement membrane and astrocyte endfeet. Due to the distinct composition of these two basement membranes, molecules regulating leukocyte migration across the BBB endothelium are distinct from the ones involved at the glia limitans, which requires expression of MMPs (matrix metalloproteinases) (Agrawal et al., 2006; Toft-Hansen et al., 2006). MMP inhibition has been associated with leukocyte retention within the perivascular space and limiting clinical symptoms (Toft-Hansen et al., 2006). Similarly, decreased MMP-2 and MMP-9 activity by inhibition of extracellular MMP inducer is correlated with higher perivascular cuff density and decreased EAE (experimental autoimmune encephalomyelitis) severity (Agrawal et al., 2012). These data emphasize the importance of leukocyte migration into the CNS parenchyma during neuroinflammatory diseases and suggest that MMPs are potential therapeutic targets to minimize disruption of the glia limitans. Nevertheless, intrinsic mechanisms are already in place to limit MMP activity. Four TIMPs (tissue inhibitors of MMPs) inhibit MMP proteolytic activity by interacting with their Zn-binding motif (Brew et al., 2000). Whereas TIMP-2, -3 and -4 are constitutively expressed within the CNS, TIMP-1 is induced upon inflammatory stimuli (Gardner and Ghorpade, 2003) including EAE and viral CNS infections (Pagenstecher et al., 1998; Khuth et al., 2001; Zhou et al., 2005b). An imbalance in the MMP/TIMP ratio is associated with several neuroinflammatory disorders (Gardner and Ghorpade, 2003). This is evidenced by decreased disease severity after induction of EAE in transgenic mice with constitutive TIMP-1 expression in the CNS, consistent with leukocyte retention within the perivascular space (Althoff et al., 2010). Similarly, T cell recruitment into the CNS parenchyma is increased in TIMP-1 deficient (TIMP-1^−/−^) mice infected with *Toxoplasma gondii* (Clark et al., 2011). Altogether, these data suggest that TIMP-1 can control leukocyte recruitment into the CNS parenchyma by regulating MMP activity at the glia limitans.

Following CNS infection with the non-fatal neurotropic MHV (mouse hepatitis virus) strain JHMV, a rapid and well-defined array of cytokines, chemokines and MMPs regulate immune cell migration into the CNS parenchyma (Bergmann et al., 2006). After initial infiltration of innate immune cells [i.e. NK (natural killer) cells, neutrophils and monocytes], recruitment of adaptive immune effectors is necessary to control virus replication and protect the host. Both CD4^+^ and CD8^+^ T cells participate in viral clearance, as well as tissue damage (Bergmann et al., 2001; Savarin et al., 2008; Stohlman et al., 2008). CD4^+^ T cells provide help for CD8^+^ T cell survival and optimal anti-viral functions within the CNS (Zhou et al., 2005a; Phares et al., 2012). However, CD4^+^ and CD8^+^ T cells display differential migration patterns. Whereas CD8^+^ T cells are directly recruited into the CNS parenchyma after crossing the BBB, CD4^+^ T cells transiently accumulate within the perivascular space before trafficking into the parenchyma (Stohlman et al., 1998). Previous analysis of MMP and TIMP expression demonstrated unique TIMP-1 up-regulation within the CNS during JHMV infection with no increased expression of TIMP-2, -3 and -4 (Zhou et al., 2005b). In contrast to other models of CNS inflammation (i.e. *T. gondii* infection and EAE), TIMP-1 was not upregulated in astrocytes but was specifically expressed by CD4^+^ T cells confined to the perivascular space, with rare expression in CD4^+^ T cells within the CNS parenchyma (Zhou et al., 2005b), suggesting that TIMP-1 delays CD4^+^ T cell migration across the glia limitans.

The present study thus aimed to determine the role of TIMP-1 in regulating CD4^+^ T cell recruitment into the CNS parenchyma, as well as potential functional consequences of altered CD4^+^ T cell distribution on JHMV induced encephalomyelitis. Although virus clearance was not altered in the absence of TIMP-1, disease severity was decreased between day 7 and 12 p.i. (post-infection), correlating with decreased IFNγ (interferon γ) production. However, flow cytometric analysis showed no difference in overall CNS leukocyte infiltration comparing WT and TIMP-1^−/−^ mice. Surprisingly, immunohistochemistry revealed that TIMP-1 deficiency correlated with increased rather than decreased CD4^+^ T cell accumulation in perivascular cuffs. No alterations in the expression of other TIMPs, MMPs or chemokines were observed in infected TIMP-1^−/−^ mice, suggesting the absence of compensatory mechanisms. In addition, increased MMP9 activity suggests during acute viral encephalomyelitis, TIMP-1 facilitates CD4^+^ T cell migration into the CNS parenchyma in a MMP-independent manner.

## MATERIALS AND METHODS

### Mice and virus

Homozygous TIMP-1 deficient (TIMP-1^−/−^) mice on the C57BL/6 background were kindly provided by Dr P.D. Soloway (Cornell University, Ithaca, NY, U.S.A.) (Lee et al., 2005) and bred locally. C57BL/6 control mice were purchased from the National Cancer Institute (Frederick, MD, U.S.A.). The study was carried out in accordance with the recommendations in the National Institute of Health Guide for the Care and Use of Laboratory Animals. All procedures were performed in compliance with the Cleveland Clinic Institutional Animal Care and Use Committee approved protocol number 2011-0553 and all efforts were made to minimize animal suffering. Mice were infected i.c. (intracerebrally) at 6 to 7 weeks of age with 1000 pfu (plaque-forming units) of the glia tropic JHMV-neutralizing mAb (monoclonal antibody)-derived 2.2v-1 variant (Fleming et al., 1986). Clinical disease severity was graded daily according to the following scale: 0, healthy; 1, hunched back and ruffled fur; 2, partial hind limb paralysis or inability to maintain the upright position; 3, complete hind limb paralysis, 4; moribund or dead. CNS virus titers were determined by plaque assay from clarified homogenates of individual mice as previously described (Stohlman et al., 2008). Briefly, brains were homogenized in Dulbecco's PBS using TenBroeck tissue grinders. Following clarification by centrifugation at 400×***g*** for 7 min at 4°C, supernatants were stored at −70°C until infectious virus was determined by plaque assay using a murine astrocytoma cell line.

### Isolation of CNS-derived cells and flow cytometry

After centrifugation of brain homogenates, cell pellets were resuspended in RPMI 1640 medium supplemented with 25 mM HEPES (pH 7.2) and adjusted to 30% Percoll (Pharmacia). A 1 ml 70% Percoll underlay was added prior to centrifugation at 800 ***g*** for 30 min at 4°C. Cells were recovered from the 30/70% interface and washed with the RPMI medium. CNS-derived cells were then resuspended in FACS buffer (PBS containing 0.1% (w/v)BSA) and non-specific binding inhibited by incubation with mouse serum and anti-mouse FcγIII/II mAb for 15 min on ice. For surface staining, cells were incubated with anti-CD45 (clone Ly-5), anti-Ly6G (clone 1A8), anti-CD11b (clone M1/70), anti-F4/80 (Serotec), anti-CD4 (clone GK1.5), anti-CD8 (clone 53-6.7) and anti-I-A/I-E (clone 2G9) (all from BD Biosciences; except when indicated) for 30 min on ice. Cells were washed twice with FACS buffer and fixed with 2% (v/v) PFA (paraformaldehyde) prior analysis. Samples were analyzed using a FACS Calibur flow cytometer (BD Biosciences) and FlowJo Software (TreeStar Inc.).

### Histology

After ice-cold PBS perfusion followed by 4% PFA, brains were dissected, fixed in 4% PFA for 1 h and incubated in 15% (w/v) sucrose for 30 min at room temperature (20°C), 20% sucrose for 30 min at 4°C and 30% sucrose overnight at 4°C. Tissues were stored in cryoprotection solution until 30 μm sections were prepared using a sliding microtome (Leica Microsystems). Sections were treated with 1% (v/v) Triton X-100 followed by blocking solution for 30 min each, and then stained with rabbit anti-mouse laminin (Cedarlane Laboratories, Ontario, Canada) and rat anti-mouse CD4 (BD Pharmingen) overnight at 4°C. Sections were then incubated with Alexa Fluor 488 goat anti-rat (Invitrogen) and Alexa Fluor 594 goat anti-rabbit (Invitrogen) secondary Abs for 1 h at room temperature, mounted with Vectashield mounting media (Vector Laboratories) and analyzed using a Leica SP5 confocal microscope. For CD4^+^ T cell quantification, brains (at least three per group) were embedded in Tissue-Tek OCT (Sakura Finetex), flash frozen in liquid nitrogen, and stored at −70°C until 10 μm sagittal sections of whole brain were prepared using a Thermo Shandon cryostat. Sections were fixed for 10 min in cold acetone. Distribution of CD4^+^ T cells was determined by immunoperoxidase staining with Vectastain ABC immunoperoxidase kit (Vector Laboratories) using purified rat anti-mouse CD4 (BD Pharmingen) as the primary Ab, mouse adsorbed rabbit anti-rat IgG (Vector Laboratories) as the secondary Ab and NovaRED (Vector Laboratories) as the peroxidase chromogen. Stained tissue sections with hematoxylin counterstain were evaluated for distribution of CD4^+^ T cells in the perivascular space and within the parenchyma in three 10× fields (cerebrum, brain stem and cerebellum). Counts from the three regions were totaled and expressed as percentage perivascular CD4/total CD4. For analysis of demyelination, spinal cords were fixed in 10% (w/v) zinc formalin, divided in six sections (from cervical, thoracic and lumbar regions) and embedded in paraffin. Sections were stained with LFB (Luxol Fast Blue) to quantify areas of demyelination within the white matter tracks. Sections at all six levels were analyzed in a blinded manner, scanned with an Aperio ScanScope (Vista) at 40× and digitally imaged at high resolution. The percentage of myelin loss within white matter areas was quantified using Aperio Software.

### Gene expression analysis

RNA was extracted from individual brains homogenized in TRIzol reagent (Invitrogen) according to the manufacturer's instructions, DNase treated and reverse transcribed as previously described (Phares et al., 2011). Semi-quantitative gene expression was analyzed using a 7500 Fast Real-Time PCR system (Applied Biosystems), SYBR green master mix (Applied Biosystems) and the following primers: CXCL10: F: 5′- GACGGTCCGCTGCAACTG-3′, R: 5′-GCTTCCCTATGGCCCTCATT-3′; CCL5: F: 5′- GCAAGTGCTCCAATCTTGCA-3′, R: 5′-CTTCTCTGGGTTGGCACACA-3′; MMP3: F: 5′-TTTAAAGGAAATCAGTTCTGGGCTATA-3′, R: 5′-CGATCTTCTTCACGGTTGCA-3′; MMP12: F: 5′-GGAGCTCACGGAGACTTCAACT-3′, R: 5′-CCTTGAATACCAGGTCCAGGATA-3′; TIMP1: F: 5′-CCAGAGCCGTCACTTTGCTT-3′, R: 5′-AGGAAAAGTAGACAGTGTTCAGGCTT-3′; TIMP2: F: 5′-ACGCTTAGCATCACCCAGAAG-3′, R: 5′-TGGGACAGCGAGTGATCTTG-3′; TIMP3: F: 5′-ATCCCCAGGATGCCTTCTG-3′, R: 5′-CCCTCCTTCACCAGCTTCTTT-3′; GAPDH: F: 5′- TGCACCACCAACTGCTTAG -3′, R: 5′- GGATGCAGGGATGATGTTC-3′. TaqMan primers and 2X Universal TaqMan Fast Master Mix (Applied Biosystems) were used to analyze IFNγ and TIMP-4 mRNA. Transcript levels were normalized to the housekeeping gene GAPDH and converted to a linearized value using the formula [2(C_T_GAPDH–C_T_gene)]×10^3^, where C_T_ is the threshold cycle value.

### ELISA

CCL5 and CXCL10 protein levels were measured on brain supernatant using quantikine ELISA according to manufacturer instructions (R&D Systems).

### Zymography

2.5×10^5^ cells isolated from the CNS as described above were resuspended in lysis buffer (1% Triton X-100, 300 mM NaCl, 50 mM Tris, pH 7.4). Lysates were then separated on 10% (w/v) acrylamide gels containing 1% (w/v) gelatin (Bio-Rad). After electrophoresis, gels were incubated in 1× renaturing buffer (Bio-Rad) for 30 min at room temperature, 1× developing buffer (Bio-Rad) for 20 min at room temperature, and overnight incubation at 37°C. Gels were then stained in 0.25% (w/v) Coomassie Brilliant Blue R-250 (Bio-Rad) and destained with the destain solution (Bio-Rad) until bands appeared.

### Statistical analysis

Data represent the means±S.E.M. and statistics were calculated using a two-way ANOVA with bonferroni post-test. *P* values <0.05 were considered statistically significant. Graphs were plotted using GraphPad Prism 4.0c software.

## RESULTS

### TIMP-1 deficiency decreases clinical disease without altering virus clearance

**Figure 1:**
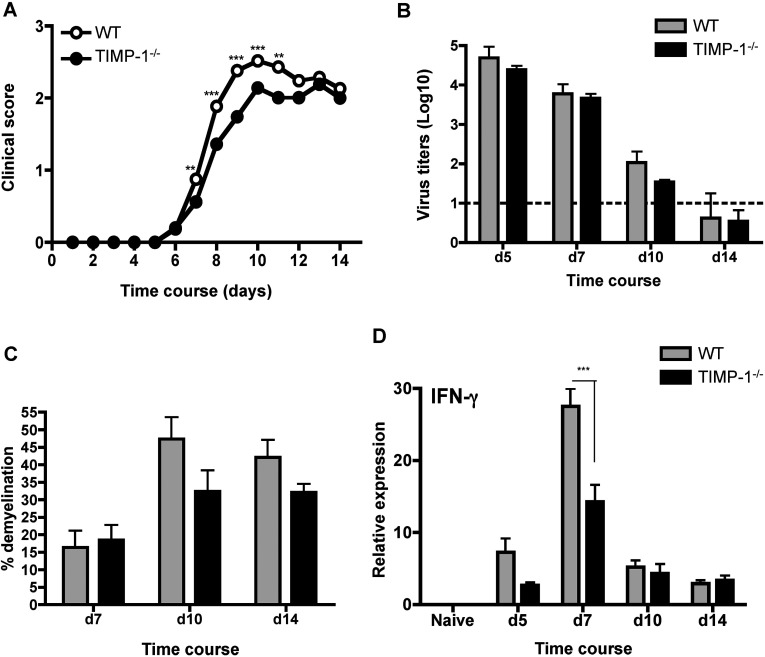
Disease severity is decreased in TIMP-1^−/−^ compared to WT mice, despite similar virus clearance and extent of demyelination (**A**) Clinical symptoms were monitored daily in WT and TIMP-1^−/−^ mice. Data represent the mean of seven experiments with 15–20 mice per experiment, **P<0.01 and ***P<0.001. (**B**) Virus replication in brains of WT and TIMP-1^−/−^ mice analyzed by plaque assay. Data represent the average±S.E.M. of six individual mice per group per time point combined from two experiments. (**C**) Percentage of demyelination in spinal cord white matter of WT and TIMP-1^−/−^ mice between days 7 and 14 p.i. Data represent the mean±S.E.M. of at least three mice per group per time point. (**D**) IFNγ mRNA expression analyzed by real-time PCR in naïve, JHMV infected WT and TIMP-1^−/−^ mice. Data represent the mean±S.E.M. of three individuals per group per time point.

To determine a role of TIMP-1 in regulating CD4^+^ T cell access to the CNS parenchyma, as well as functional consequences, WT (wild-type) and TIMP-1^−/−^ mice were infected with the gliatropic JHMV strain of MHV. Signs of encephalitis, characterized by hunched back and ruffled fur, were initially observed around day 6 p.i. with no differences between WT and TIMP-1^−/−^ mice (Figure 1A). However, as symptoms progressed throughout ~day 10 p.i., disease severity was significantly decreased in infected TIMP-1^−/−^ compared to WT mice (Figure 1A). Nevertheless, reduced clinical symptoms were only transient as both groups displayed similar disease severity after day 12 p.i. (Figure 1A).

Disease severity during JHMV infection is not only dependent on viral load, but also the balance of T cell activities, especially IFNγ secretion (Kapil et al., 2009; Phares et al., 2010). The complexity of these interactions is highlighted by studies in mice with altered T cell activities: reduced clinical disease despite similar virus load, coincided with reduced IFNγ in IL-12 p35 subunit deficient mice (Kapil et al., 2009), while more severe clinical symptoms, despite accelerated virus control coincident with increased IFNγ in B7-H1 deficient mice (Phares et al., 2010). The participation of both CD4^+^ and CD8^+^ T cells in JHMV clearance and tissue damage (Bergmann et al., 2001; Stohlman et al., 2008) and the helper role of CD4^+^ T cells provided to anti-viral CD8 T cells within the CNS (Stohlman et al., 1998; Phares et al., 2012) constitute additional layers of complexity. Finally, the finding that retention of T cells within the perivascular space is associated with delayed control of JHMV replication and disease onset (Savarin et al., 2010) supports the importance of parenchymal T cell localization in determining pathogenic outcome. Thus, transiently decreased disease severity in TIMP-1^−/−^ mice may be explained by accelerated parenchymal CD4^+^ T cell infiltration mediating accelerated local T cell activity and virus control. However, similar peak virus replication at day 5 p.i., and decline of virus titers at day 7 p.i. with a drop below detection levels by day 14 p.i. in both groups, demonstrated that control of virus replication was not altered by the absence of TIMP-1 (Figure 1B). Similarly, reduced disease severity did not correlate with decreased myelin loss in TIMP-1^−/−^ compared to WT mice, as extent of demyelination was similar comparing the two groups at days 7, 10 and 14 p.i (figure 1C).

However, analysis of a potential correlation between decreased disease severity and IFNγ, revealed that IFNγ mRNA was reduced in the CNS of TIMP-1^−/−^ compared to WT mice at days 5 and 7 p.i. (Figure 1D). No differences in IFNγ were noted at later times p.i. (Figure 1D) when virus replication was already significantly reduced. These data demonstrated that limited early IFNγ levels within the CNS were associated with prolonged attenuated clinical disease in TIMP-1^−/−^ mice. Moreover, reduced IFNγ implied that local T cell stimulation by viral antigen was specifically reduced in the absence of TIMP-1 at the time of maximal T cell migration to the CNS.

### TIMP-1 does not regulate overall CNS leukocyte recruitment

**Figure 2:**
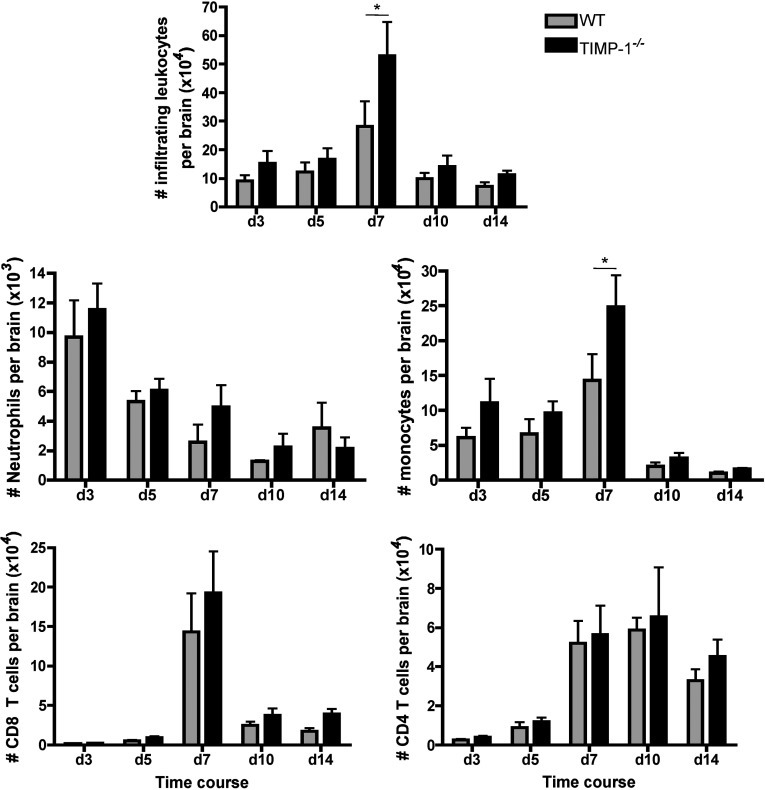
TIMP-1 deficiency does not alter CNS inflammation Numbers of total inflammatory leukocytes (CD45^hi^), neutrophils (ly6G^+^), monocytes (F4/80^+^), CD8^+^ and CD4^+^ T cells per brain in WT and TIMP-1^−/−^ infected mice. Data represent the mean±S.E.M. of 12 mice per group per time point combined from four separate experiments (*n*=3 per time point and group per experiment).

Reduced IFNγ production during the peak of antigen specific T cell stimulation within the CNS, suggested limited overall T cell recruitment and/or limited T cell access to viral antigen. Although IFNγ is mainly produced by T cells during JHMV infection, IFNγ mRNA expression is more prominent in CD4^+^ compared to CD8^+^ T cells at the population level *in vivo* (Phares et al., 2010), suggesting that decreased CNS IFNγ production in TIMP-1^−/−^ mice was mainly due to limited CD4^+^ T cell responses. However, this contradicts our initial hypothesis that the absence of TIMP-1 would facilitate CD4^+^ T cell access into, and thereby their effector function, within the CNS parenchyma. Thus, to initially verify that TIMP-1 does not affect overall leukocyte recruitment into the CNS during JHMV infection, infiltrating CD45^hi^ cells were measured by flow cytometry (Figure 2). Although CD45^hi^ leukocytes were higher at day 7 p.i. in the CNS of TIMP-1^−/−^ mice compared to controls, no differences was noted at any other time point (Figure 2). The composition of leukocyte infiltrates was also not significantly altered by the absence of TIMP-1. CNS neutrophil infiltration followed similar kinetics, with a peak at day 3 p.i. and a slight, but not significant, increase in TIMP-1^−/−^ compared to WT mice (Figure 2). This modest increase in neutrophils was sustained to day 10 p.i. The number of CNS monocytes in TIMP-1^−/−^ mice was also slightly increased at early time points, with the largest difference observed at day 7 p.i. (Figure 2). Increased CD45^hi^ leukocyte infiltration at day 7 p.i. in TIMP-1^−/−^ mice thus coincided with a relative increase in monocyte recruitment, although they decreased to similarly low levels after day 10 p.i. in both groups (Figure 2). Importantly, neither CD8^+^ nor CD4^+^ T cell recruitment was altered by TIMP-1 deficiency, as both populations peaked at almost identical levels between days 7–10 p.i. as in WT mice (Figure 2). There were also no differences in virus-specific CD8^+^ T cell recruitment (results not shown), consistent with similar virus clearance between both groups (Figure 1B). Altogether, these data suggest that TIMP-1 only exerts a minor influence on acute inflammation and does not regulate the overall composition of leukocytes recruited into the CNS following JHMV infection.

### CD4^+^ T cells are retained in the perivascular space of TIMP-1^−/−^ mice

**Figure 3:**
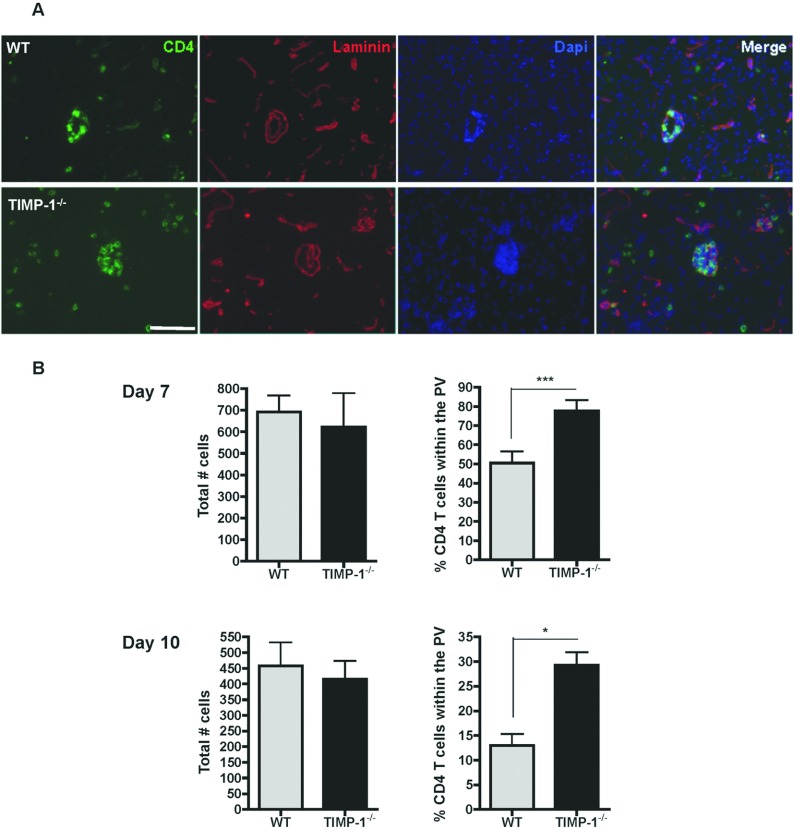
CD4^+^ T cells are retained in the perivascular space of TIMP-1^−/−^ mice (**A**) CD4^+^ T cell perivascular cuffs in WT and TIMP-1^−/−^ mice at day 7 p.i. analyzed by immunofluorescent microscopy. The two basement membranes of the BBB stained with laminin (red), delimit the perivascular space. CD4^+^ T cells appear in green. Scale bar, 25 μm. (**B**) Total number of CD4^+^ T cells and their distribution in the perivascular space versus parenchyma analyzed in WT and TIMP-1^−/−^ mice at day 7 and 10 p.i. Data represent the mean±S.E.M. of six mice per group combined from two separate experiments (with *n*=3 for each individual experiment).

Specific TIMP-1 expression by CD4^+^ T cells, coincident with their prolonged retention in the perivascular space at day 7 p.i. (Zhou et al., 2005b), initially suggested that TIMP-1 delays CD4^+^ T cell access to the CNS parenchyma by inhibiting MMP-dependent glia limitans disruption. However, decreased IFNγ, despite similar CD4^+^ T cell recruitment, suggested reduced CD4^+^ T cell effector function potentially due to impaired access to viral antigen presenting target cells in the parenchyma. This notion was tested by immunohistologic analysis of the distribution of CD4^+^ T cells in the perivascular space versus parenchyma (Figure 3A). Total numbers of CD4^+^ T cells were similar in the brain of WT and TIMP-1^−/−^ mice at day 7 and 10 p.i. (Figure 3B), consistent with flow cytometric analysis. However, CD4^+^ T cell retention within the perivascular space was nearly increased by 30% in TIMP-1^−/−^ mice at day 7 p.i. (Figure 3B). Moreover, despite the decreased extent of CD4^+^ T cell cuffs in both groups at day 10 p.i., preferential perivascular accumulation was sustained to day 10 p.i. in the absence of TIMP-1 (Figure 3B). These results clearly support a revised notion that TIMP-1 promotes, rather than limits, CD4^+^ T cell recruitment into the CNS parenchyma during JHMV induced encephalitis.

### CD4^+^ T cell retention within the perivascular space is not associated with compensation by TIMPs or altered MMP and chemokine expression

**Figure 4:**
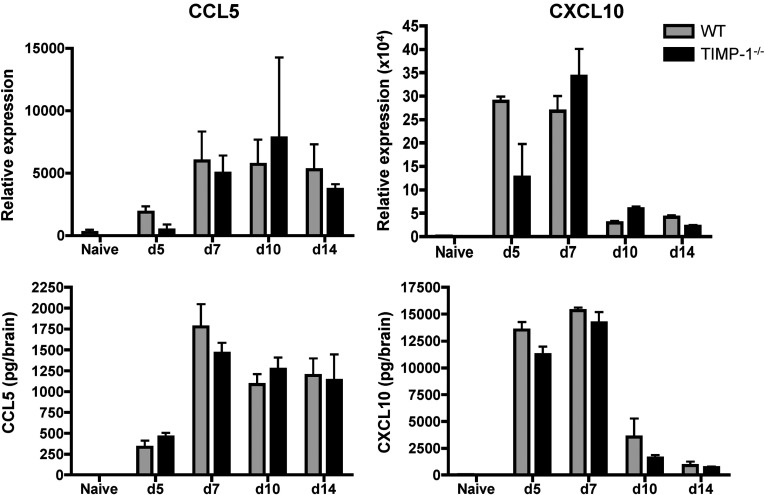
Chemokine expression in infected WT and TIMP-1^−/−^ mice CCL5 and CXCL10 mRNA, measured by real-time PCR, and protein, measured by ELISA, in naïve or infected WT and TIMP-1^−/−^ mice. Data represent the mean±S.E.M. of three to seven individual mice per group per time point.

While decreased accumulation of CD4^+^ T cells in the parenchyma was consistent with reduced antigen encounter and thus IFNγ expression, the results were inconsistent with a T cell retaining role of TIMP-1 noted in other models (Althoff et al., 2010; Clark et al., 2011). We therefore investigated whether accumulation of CD4^+^ T cells in the perivascular space of TIMP-1^−/−^ mice correlated with dysregulated chemokine and/or MMP/TIMP expression. However, we could not detect significant differences in expression of CXCL10 or CCL5 mRNA or protein, two major chemoattractants of T cells during JHMV infection (Stiles et al., 2006; Stiles et al., 2009), comparing the two groups at day 7 p.i. (Figure 4), when TIMP-1^−/−^ CD4^+^ T cells accumulate within the perivascular space.

**Figure 5:**
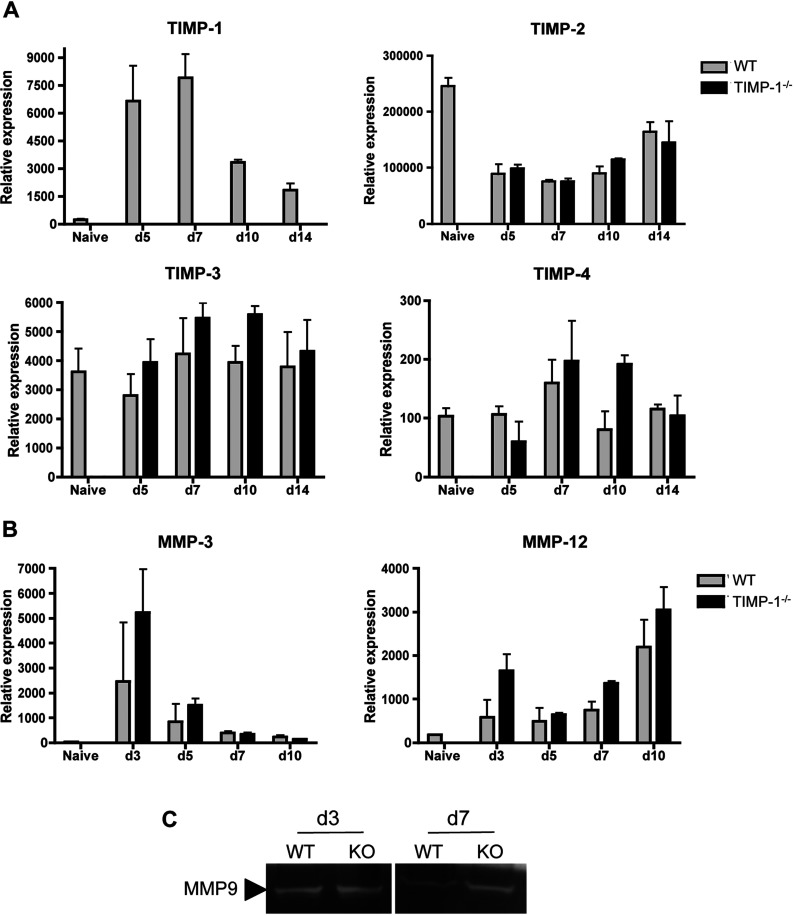
TIMPs and MMPs do not compensate for TIMP-1 deficiency during JHMV infection Expression of TIMP-1, -2, -3 and -4 mRNA (**A**), and MMP-3 and -12 mRNA (**B**), analyzed by real-time PCR in naïve and JHMV infected WT and TIMP-1^−/−^ mice. Transcript levels depict the average±S.E.M. of three individual brains per group per time point representative of two separate experiments. (**C**) MMP9 activity determined by zymography of CNS mononuclear cells isolated from infected WT and TIMP-1^−/−^ mice at days 3 and 7 p.i. Data are representative of two experiments.

The study of MMP/TIMP functions using gene deficient animals demonstrated that the lack of a single molecule is often counteracted by compensatory expression of similarly functioning proteins *in vivo* (Dubois et al., 1999; Savarin et al., 2011). To determine if TIMP-1 deficiency altered other components associated with T cell trafficking, expression of MMP inhibitors in the CNS was evaluated in both groups. Naïve mice express TIMP-1 mRNA at very low levels, but the levels increased by ~100-fold following JHMV infection (Figure 5A). Moreover, peak Timp-1 mRNA at day 7 p.i. correlates with CNS CD4^+^ T cell recruitment (Figure 2). As expected, TIMP-1 mRNA was undetectable in TIMP-1^−/−^ mice (Figure 5A). TIMP-2, -3 and to a lesser extent TIMP-4 mRNA were constitutively expressed at high levels within the CNS of naïve WT mice, but were not further up-regulated by infection. Similar to the down regulation of TIMP-2 mRNA expression following activation *in vitro* (Zhou et al., 2005b), TIMP-2 mRNA expression in the CNS was reduced after JHMV infection (Figure 5A); however no differences were observed comparing WT and TIMP1^−/−^ mice (Figure 5A). Furthermore, stable TIMP-3 and -4 mRNA expression after JHMV infection of WT mice was also not altered by the absence of TIMP-1 (Figure 5A). These data suggested that neither TIMP-2, -3 or -4 compensate for the absence of TIMP-1 following JHMV infection.

As MMPs regulate leukocyte migration through the glia limitans (Toft-Hansen et al., 2006), potential alterations of MMP expression or activity was also considered as a mechanisms underlying CD4^+^ T cell retention in the perivascular space of TIMP-1^−/−^ mice. Only a restricted number of MMPs are induced following JHMV infection, including MMP-3 and -9 by astrocytes and neutrophils, respectively, as well as MMP-12 by multiple cell types (Zhou et al., 2005b). The absence of TIMP-1 did not alter the kinetics or levels of MMP-3 and -12 mRNA expression (Figure 5B). Furthermore, MMP-9 activity, measured by zymography, was similar in both groups at day 3 p.i. (Figure 5C), consistent with identical neutrophil infiltration (Figure 2). At day 7 p.i. TIMP-1^−/−^ mice displayed increased MMP-9 activity (Figure 5C), supporting a role of TIMP-1 as a major inhibitor of MMP9 activity in WT mice (Roderfeld et al., 2007). Nevertheless, accumulation of CD4^+^ T cells in the perivascular space of TIMP-1^−/−^ mice, despite increased MMP9 activity, suggests that CD4^+^ T cell retention is MMP independent. Thus, decreased migration of CD4^+^ T cells into the CNS parenchyma in the absence of TIMP-1 was not associated with dysregulation of chemokine, MMP or TIMP expression, suggesting an MMP-independent role of TIMP-1 during JHMV infection.

## DISCUSSION

Migration of leukocytes into the CNS parenchyma requires penetration of the endothelial layer as well as the glia limitans. This two-step process is highly regulated at several distinct levels involving, integrins, chemokines and MMPs (Owens et al., 2008). Moreover, different infiltrating cell types appear to contribute to these migration processes by distinct mechanism, e.g. preferential chemokine responsiveness or MMP/TIMP release. A common observation in many neuroinflammatory disorders is the distinct migration pattern of CD4^+^ and CD8^+^ T cells through the BBB (Stohlman et al., 1998; Siffrin et al., 2009; Ploix et al., 2011). Whereas CD8^+^ T cells traverse into the CNS parenchyma efficiently, CD4^+^ T cells appear to be transiently retained within the perivascular space. This pattern is clearly demonstrated during JHMV encephalomyelitis, in which both CD4^+^ and CD8^+^ T cells are essential in mediating virus clearance accompanied by tissue damage (Bergmann et al., 2006). Transient retention of CD4^+^ T cells within the perivascular space raises both the question of its functional relevance, as well as the identification of molecules regulating T cell access to the parenchyma. During JHMV infection TIMP-1 is specifically expressed by CD4^+^ T cells when present in perivascular cuffs, but not when located in the parenchyma (Zhou et al., 2005b). These data suggested that TIMP-1 might control CD4^+^ T cell retention within the perivascular space by disrupting MMP activity at the glia limitans (Toft-Hansen et al., 2006). However, results presented herein indicate that TIMP-1 promotes rather than delays CD4^+^ T cell migration to the CNS parenchyma as evidenced by increased retention of CD4^+^ T cells around the CNS post-capillary venules in the absence of TIMP-1. Furthermore, similar expression patterns of chemokines and MMPs and TIMPs in TIMP-1^−/−^ and WT mice, support an MMP independent function of TIMP-1 in regulating CD4^+^ T cell access to the CNS parenchyma.

TIMP-1 was initially characterized as an inhibitor of MMP activity, with a primary role in remodeling the extracellular matrix (Brew et al., 2000). TIMP-1-dependent MMP inhibition is also implicated in several other biological processes, including cell growth, angiogenesis and apoptosis (Gardner and Ghorpade, 2003). However, TIMP-1 also exerts MMP independent effects (Stetler-Stevenson, 2008), i.e. apoptosis or cell proliferation, highlighting its complex and pleiotropic functions. Despite a correlation between imbalanced MMP/TIMP activity with several neuroinflammatory disorders (Gardner and Ghorpade, 2003), the role of TIMP-1 remains elusive. During EAE, constitutive TIMP-1 exerts a protective role by maintaining BBB integrity through MMP inhibition and limiting leukocyte infiltration into the CNS parenchyma (Althoff et al., 2010). During chronic EAE, TIMP-1 plays an MMP-independent role (Crocker et al., 2006), potentially by promoting oligodendrocyte progenitor differentiation and remyelination (Moore et al., 2011). While our data support an MMP independent function of TIMP-1 in promoting leukocyte migration across the glia limitans, the mechanism remains unclear. Increased proteases may inactivate function of molecules essential in CD4 T cell migration within the CNS parenchyma. Another mechanism may involve TIMP-1 interaction with the tetraspanin CD63 (Jung et al., 2006). CD63 is upregulated by activated human T cells and acts as a co-stimulatory molecule (Pfistershammer et al., 2004). Thus, restimulation of CD4^+^ T cells upon entry into the CNS might be altered in the absence of TIMP-1 and CD63 complexes, leading to accumulation of insufficiently activated CD4^+^ T cells within the perivascular space. Restimulation of CD4^+^ T cells after interaction with antigen-presenting cells within the perivascular space is a prerequisite for disease pathogenesis in EAE (Greter et al., 2005). Indeed, retention of CD4^+^ T cells within the perivascular space of JHMV infected TIMP-1^−/−^ mice also correlated with decreased disease severity and IFNγ production. Whether decreased IFNγ is a consequence of reduced antigen presenting cell-T cell activation in the perivascular space or limited T cell access to parenchymal antigen presenting cells remains unclear. Nevertheless, CD4^+^ T cells are potent producers of IFNγ during JHMV infection (Phares et al., 2010) and both IFNγ and CD4^+^ T cells contribute to disease severity (Kapil et al., 2009; Phares et al., 2012). IFNγ activates microglia/macrophages, which enhance disease severity during JHMV infection (Savarin et al., 2010) as well as other neuroinflammatory diseases (Ajami et al., 2011).

CD4^+^ T cells also promote virus-specific CD8^+^ T cell responses both during initial peripheral activation and at the effector site during JHMV infection (Stohlman et al., 1998; Phares et al., 2012). Nevertheless, CD4^+^ T cell accumulation within the perivascular space did not affect control of virus replication, suggesting that CD8^+^ T cells, primary mediators of JHMV control, were fully functional in the absence of TIMP-1. CD4^+^ T cell helper function to CD8^+^ T cells thus appeared to be preserved despite altered CD4^+^ T cell distribution in the absence of TIMP-1.

Finally, the difference between the MMP-independent function of TIMP-1 during JHMV infection and its role in limiting leukocyte access through the BBB by inhibiting MMPs in other neuroinflammatory models (Althoff et al., 2010; Clark et al., 2011) may correlate with the cellular source of TIMP-1. Indeed, TIMP-1 is specifically expressed by CD4^+^ T cells during JHMV infection with no parenchymal expression (Zhou et al., 2005b). By contrast, TIMP-1 is up-regulated in astrocytes during both EAE (Pagenstecher et al., 1998) and toxoplasma infection (Clark et al., 2011), and also limits T cell access to the CNS parenchyma. Astrocyte endfeet are part of the glia limitans and are adjacent to the parenchymal basement membrane, which is disrupted by MMPs (Toft-Hansen et al., 2006). Thus, astrocytic secretion of TIMP-1 may directly inhibit MMP activity at the glia limitans because of their close proximity. By contrast, during JHMV infection, TIMP-1 may have limited access to the enzymatic site of MMPs and act in an autocrine fashion (Oelmann et al., 2002).

In summary, our data demonstrate a novel MMP-independent role of TIMP-1 in regulating CD4^+^ T cell migration across the glia limitans during viral encephalitis. Whereas reduced CD4^+^ T cell access to the CNS parenchyma did not affect viral control, disease severity was reduced. Thus, targeting TIMP-1 may represent a potential therapeutic target to reduce pathogenesis, without altering virus clearance, during viral encephalomyelitis.
